# Global research challenges and opportunities for mental health and substance-use disorders

**DOI:** 10.1038/nature16032

**Published:** 2015-11-19

**Authors:** Florence Baingana, Mustafa al’Absi, Anne E. Becker, Beverly Pringle

**Affiliations:** 1 Makerere University School of Public Health, PO Box 7072, Kampala, Uganda.; 2 Duluth Medical Research Institute (DMRI), University of Minnesota Medical School, 311-1035 University Drive, Duluth, Minnesota 55812, USA.; 3 Department of Global Health and Social Medicine, Harvard Medical School, 641 Huntington Avenue, Boston, Massachusetts 03115, USA.; 4 Office for Research on Disparities & Global Mental Health, National Institute of Mental Health, 6001 Executive Boulevard, Room 7207, Bethesda, Maryland 20892, USA.

## Abstract

The research agenda for global mental health and substance-use disorders has been largely driven by the exigencies of high health burdens and associated unmet needs in low- and middle-income countries. Implementation research focused on context-driven adaptation and innovation in service delivery has begun to yield promising results that are improving the quality of, and access to, care in low-resource settings. Importantly, these efforts have also resulted in the development and augmentation of local, in-country research capacities. Given the complex interplay between mental health and substance-use disorders, medical conditions, and biological and social vulnerabilities, a revitalized research agenda must encompass both local variation and global commonalities in the impact of adversities, multi-morbidities and their consequences across the life course. We recommend priorities for research – as well as guiding principles for context-driven, intersectoral, integrative approaches – that will advance knowledge and answer the most pressing local and global mental health questions and needs, while also promoting a health equity agenda and extending the quality, reach and impact of scientific enquiry.

The global mental health landscape has transformed over the past 25 years because of the higher visibility of the burden of mental health and substance-use disorders^1^. These disorders comprise 7.4% of global disability-adjusted life years (DALYs) and 22.7% of global years lived with disability (YLDs)^2^ (Supplementary Information)^3^. The main contributors worldwide are depression and dysthymia (9.6% of all YLDs); anxiety (3.5% of all YLDs); and schizophrenia, substance-use disorders and bipolar disorder (just over 2% of all YLDs). Alcohol and substance-use disorders come in second for most of the developing world, more so for southern Africa (drug use) and Eastern Europe (alcohol)^2^. The burden of mental health and substance-use disorders is predicted to increase worldwide in coming decades, and the steepest rise can be expected in low- and middle-income countries (LMICs) as a result of rising life expectancy, population growth and under-resourced health care^4^. For example, simulation models predict a 130% increase in associated health burden of alcohol and substance misuse in sub-Saharan Africa by 2050 as a result of population growth and ageing^5^. As substantial as they are, conventional health metrics do not capture additional social burdens attached to living with mental illness. Untreated mental health disorders are associated with a high economic burden^6^. Furthermore, pervasive stigma and human rights violations compound the suffering associated with these disorders and exacerbate social vulnerabilities^7-9^.

As the health, social, economic and human costs of mental and substance-use disorders become increasingly better documented, political will and multilateral commitments to scale up mental health care in LMICs have grown. The World Health Organization has introduced a series of policy initiatives that articulate both high-level aspirations and pragmatic guidance for mental health and substance-use services delivery in LMICs. The most recent, the Global Mental Health Action Plan 2013-2020, challenges member states, partners and the Secretariat to collectively meet ambitious goals by the year 2020, including increasing mental health care coverage by 20% for severe mental health illness and reducing national suicide rates by 10% (ref. 10). A consideration of the timeline of these landmark events – including the roll out of a number of key funding and policy initiatives that target the persisting resource gaps – illustrates the substantial momentum in integrating mental health into the broader global health agenda that has occured over the past few decades (See Supplementary Information).

An interactive map, depicting the broad geographical distribution of current, promising initiatives in global mental health is available at http://www.nimh.nih.gov/about/organization/gmh/global-research-on-mental-health-and-substance-abuse.shtml. Policy and programmatic initiatives have laid a foundation for strengthened global mental health services by developing an initial consensus scientific agenda that focuses energies and funding on the most crucial research for building an empirical base. Key funding initiatives have supported research to leverage scarce resources and improve access through task sharing, integration of mental health care into existing primary health-care infrastructure and enhancement of diagnostic assessment. Increasingly, resources have been allocated to build in-country research capacities and strengthen collaboration through institutional partnerships^11^. Complementary graduate-level training programmes in global mental health have also emerged, although mental health specialty training, as a track, remains relatively under-represented among other global health domains^12,13^.

## Key research gaps and challenges

The global health burden of mental health disorders is exacerbated by the growing concurrent problems associated with substance misuse. Substance use and exposure to addictive drugs have chronic and profound effects on neurobehavioural and neurodevelopmental functions. In LMICs, the socioecology of poverty, malnutrition, political conflicts and poor health systems influence the epidemiology, as well as the adverse outcomes, that result from substance misuse. Additional challenges associated with co-morbidity stem from its augmentation of clinical burden, through increased risk for relapse, other infectious and medical complications, and economic hardship and homelessness. In this context, co-morbid substance use and mental illnesses in particular may contribute to increasing health burden. The prevalence of substance-use disorders has escalated in recent decades, reaching 5.4% of the total disease burden and 9.1% when tobacco use is included^14^.

Individuals with substance-use disorders are also likely to have other mental health problems, including depression and schizophrenia^4,15^. Similarly, a large proportion of people with mental illnesses also have substance-use disorders^16,17^. Research that investigates the relationship between mental illness and substance-use disorder has yielded mixed findings, with some support for causal relationships in both directions as well as for shared genetic, environmental, social and cultural risk factors. For example, cannabis use is linked to a risk of developing psychotic illness^18^. Conversely, mental illness may increase the risk of substance misuse; individuals may ‘self-medicate’ with alcohol, tobacco or amphetamines as a means of coping with distress and negative affects^19,20^. Some factors, including genetic vulnerabilities, traumatic *exposures and stress*, may confer risk for both conditions^21,22^. Diagnosis and treatment of co-morbid substance misuse and mental health illness remains a significant challenge, particularly in LMICs. The burden of this co-morbidity is further exacerbated by the increased clinical complexity that stems from resistance to treatment, risk of relapse, vulnerability to other infectious and medical complications, and increased economic hardship and homelessness.

**Figure 1:**
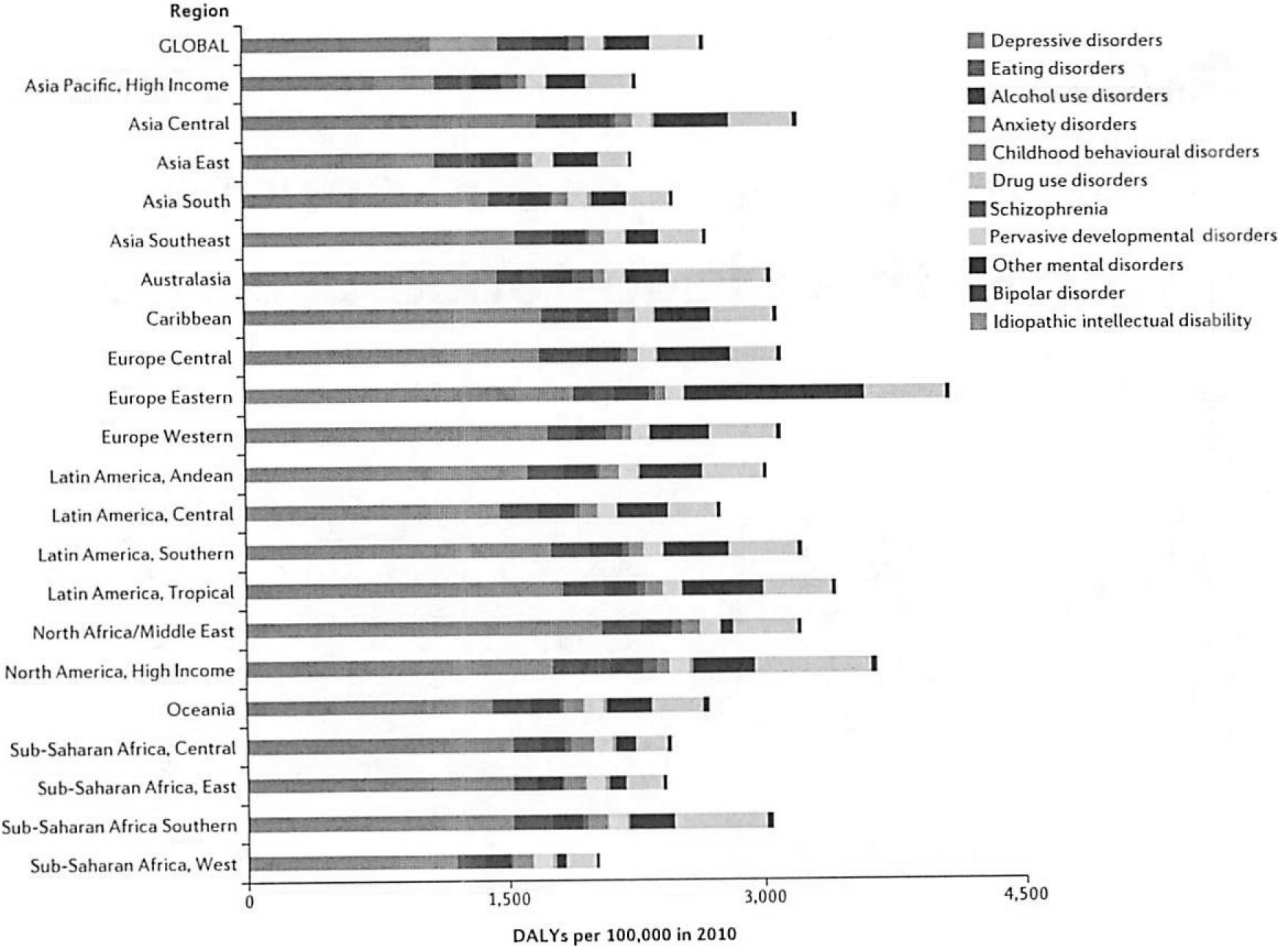
Burden of risk associated with substance-use disorders varies across the world as disability adjusted life years (DALYs). Reprinted with permission from ref.^4^.

The burden and configuration of risk associated with substance-use disorders and co-morbid mental illness seem to vary across the world (Fig. 1)^14^. Although alcohol and opioid problems are escalating in Europe, Africa and Asia, problems associated with amphetamines and cannabis are more prevalent in Asia, North America and Europe. Cocaine use is prevalent in North America and Europe, whereas misuse of indigenous psychoactive substances is prevalent in other regions, such as the use of khat in parts of Africa and the Middle East and that of coca leaves in South America^23^. Notably, existing knowledge gaps may underestimate the impact of substance-use disorders^24^. The full extent of adverse mental health and social impacts of substance-use disorders such as alcohol use during pregnancy and fetal alcohol spectrum disorders^25^ remain incompletely understood.

Mental health and substance-use disorders also frequently co-occur with other diseases, increasing associated morbidity and mortality risk^26,27^. It is not uncommon for individuals with HIV/AIDS or non-communicable diseases such as hypertension, diabetes and cardiovascular disease to also have symptoms of depression or anxiety and to use alcohol or other drugs to excess. Attention deficit hyperactivity disorder has been associated with risky sexual behaviours that can result in transmission of HIV/AIDS. These interdependent illnesses stem from common risk factors, such as childhood adversity; and bidirectional influences, such as poor treatment adherence^28^ and increased engagement in risky behaviour^29-31^. Growing awareness of the complex interplay between mental illness and the increasing burden of chronic disease globally has prompted research that examines the effects of depression on adherence to medical treatments and the effects of integrated care – co-treatment of high blood pressure and depression, for example – on the outcomes of both of the co-occurring illnesses (see for example refs 32-34). A life-course approach to risk reduction that takes into account risks that occur in childhood and early adulthood, and that promotes a healthy lifestyle, and early recognition and treatment of mental and substance-use disorders is essential to curtail the long-term negative impacts of many preventable health risks.

## TREATMENT GAP

The proportion of people who need, but do not receive care is especially high in LMICs^35,36^ The inadequate resourcing of mental health care in LMICs has been widely documented and critiqued. For example, on average less than 3% of public health resources are allocated to specific mental health care in LMICs, with even less (around 1%) in Africa and Asia^37^. Most LMICs have far fewer health-care professionals than they need to deliver mental health and substance-use interventions to everyone who needs them^38,39^. Scaling up services will require more than training additional psychiatrists, psychologists and psychiatric nurses, however, strategic leveraging of scarce resources will also be necessary. In particular, task shifting – delegating health-care tasks from specialists to various non-specialist health professionals and other health workers – has shown promise for certain mental health and substance-use interventions^40-43^. In addition, the integration of mental health services into primary health-care delivery settings through community-based and task-sharing approaches can both help to reduce burden on carers and improve access and the coordination of care. Mental health services and health-system strengthening, and in particular, task shifting, as well as organization and ways of delivering community-based mental health services in LMICs should be prioritized for research.

There is also a substantial gap in scientific knowledge for preventing and treating mental health and substance-use disorders. In addition, what is currently known is often not applicable to low-resource regions. Intervention strategies to address substance-use disorders have improved over recent decades, but have had limited success in achieving total recovery and have limited coverage in LMICs^15^. Moreover, resources for providing these interventions are constrained or lacking in most LMICs^15,24^. Models for improving availability and access to effective mental health care emphasize the integration of both prevention and treatment services within primary care systems. This has been a core approach taken by the WHO Mental Health Gap Action Program (mhGAP)^44,45^.

Most published clinical trial data on therapeutics for mental health disorders are based on research conducted in high-income countries^46^-^48^. In the absence of region-specific empirical data, deployment of these therapeutic strategies in LMICs is a reasonable pragmatic compromise in the short term when informed by local knowledge, and pending rigorous and systematic evaluation. Local research on clinical effectiveness of these treatments and implementation research on how to deliver these therapies and scale them up are urgently needed.

### Priorities for advancing the global mental health agenda

Our recommendations build on the strong base of empirical evidence and previous consensus statements and reports that have articulated principles, needs and priorities that should inform a robust research agenda (Table 1). The predominant focus of global mental health research is currently on health services and implementation research, areas that align well with efforts to close treatment gaps and that must continue to be strengthened. Whereas we regard these contributions as formative and arguably the most pragmatic and exigent in the short term, they should not pre-empt a more ambitious scope of scientific inquiry that ranges from basic sciences to health policy. Innovation should encompass much more than strategies to leverage scarce resources lest the scope of progress in the field be consigned to improving the efficiency of old models of care delivery. Instead, complementary and parallel lines of context-driven research should also aim to advance the scientific understanding of aetiology, population-specific phenotypic variation in presentation and course, and differential response to therapeutics through promising avenues in neuroscience, biomarkers, genetics and epigenomics.

## EPIDEMIOLOGY

Epidemiological research is crucial to better understand the differential risk factors and burden of mental health and substance-use disorders across diverse geographical regions and social contexts. Refinement of approaches to diagnostic assessment that are both locally valid and relatable to global classification is essential to more effective and efficient case identification, particularly in the hands of non-specialists. Such advances will generate more accurate estimates of health burdens and salient risk factors on which local health policymakers can draw. In addition, research is needed to better define the health and social impacts of syndemic mental health disorders, substance-use disorders and medical diseases, as well as to understand how social adversities moderate and mediate risk of onset, severity and course. Such research will inform optimal strategies for prevention, treatment and follow-up care for individuals with these co-morbidities.

## BASIC SCIENCE RESEARCH

The research agenda to address the global burden of mental health and substance-use disorders should build on recent advances in the field of basic neuroscience, biomarkers, proteomics, and genetics and epigenetics. For example, research in the past decade has identified molecular and structural markers connected with mental health and substance-use disorders^49^. These include protein alterations in the form of upregulation of a 40-amino acid VGF-derived peptide and the downregulation of apoAl protein in schizophrenia^50^. Hormonal and physiological alterations in stress- and appetite-related neuropeptides have also been pursued in the context of addiction and treatment outcome^51^-^53^. There has also been significant interest in epigenomics and how it could advance our understanding and use of biomarkers. Epigenomic modifications affect gene expression, and involve multiple molecular steps, including DNA methylation^54^. In light of evidence that indicates a role for epigenetic mechanisms in modifying genes that increase propensity for drug use and mental illness, it is important to develop a means by which this approach could be harnessed to improve the validity and reliability of diagnostic measures as well as to help to tailor interventions to the individual. Research that considers the diversity of environmental exposures and gene-environment interactions across different settings can advance the utility of these markers to confirm diagnosis and to predict treatment outcome. Furthermore, such markers may be useful in identifying those at high risk so that measures can be applied to prevent initial risk or onset, or slow down or prevent progression towards psychopathology. The use of such approaches should also parallel the development of conceptual models to guide understanding of the complex, multidimensional aetiology of mental health and substance-use disorders. To that end, global research that focuses on mental health and substance-use disorders should take into account how genetics and exposure to environmental toxins interact with social, cultural and environmental conditions to moderate the risk of these disorders.

## HEALTH DELIVERY AND IMPLEMENTATION RESEARCH

Four out of the top five research priorities identified in the grand challenges statement – developed by a consortium of researchers, advocates and clinicians with funding from the US National Institute of Mental Health (NIMH) and the Global Alliance for Chronic Diseases – fall in the domain of enhancing the quality of, and access to, mental health care^55^. This call to invest in health services and implementation research is in response to identified treatment gaps as well as their numerous antecedents, such as weak health systems, *shortfalls in human* and financial resources, and social structural barriers to care. There is ample evidence for science-based care and the integration of mental health services into primary health care. However, we still lack crucial knowledge on how best to disse*minate and implement evidence*-based mental health interventions in resource-poor contexts, including those characterized by the extreme social adversities associated with political conflict, displacement and destitution. Future research is therefore necessary to rigorously evaluate and optimize effectiveness of task sharing, integration of mental health into primary care, and deployment of the mhGAP algorithms at larger scale and across diverse social settings^41,56^.

Key strategies for expanding access to high-quality mental health care in LMICs come from models that are successful in leveraging scarce resources in other clinical domains. However, challenges unique to care delivery for mental health and substance-use disorders warrant special attention and innovation. These include how to improve diagnostic assessment and population health surveillance, given the heterogeneous and sometimes opaque presentations of signs and symptoms across diverse social and cultural contexts^57,58^; how to address the social and cultural factors, especially stigma, that hinder access to care and may prevent patients with mental illnesses and substance-use problems from using the resources available for prevention and treatment; how to mitigate social vulnerabilities, such as poverty *and gender-based* violence that elevate risk of mental disorders, while *building on sociocultural* resources that promote coping and resilience; how to develop coordinated approaches to strategic preventive interventions, monitoring and targeted treatments over the life course and across *disorders, given developmental* trajectories of mental health and substance-use disorders, and their harmful symbiosis with other chronic conditions and vulnerabilities; and how to rapidly scale up effective interventions to close the treatment gap in resource constrained environments^59^-^61^. Priorities for global mental health research resonate with the global health agenda, with its focus on reducing health burdens^62^. In this respect, a globalizing framework aimed at developing approaches that are effective when scaled up and implemented across geographically and socially diverse settings and populations reflect pragmatic goals of responding to pressing needs. We emphasize, however, that closing the prevailing treatment gaps for mental health and substance-use disorders will also depend on fortifying scientific inquiry so that we can understand the, sometimes remarkable, local variation in manifestation and course of mental disorders^63^.

## TRANSLATIONAL AND HEALTH-POLICY RESEARCH

Ensuring that populations receive high-quality care that improves mental health is the purview of policymakers. Shaping sound public policies that are based on up-to-date research can be challenging, but promising examples exist. An experimental housing policy called Moving to Opportunity found that moving from a high-poverty to a lower-poverty neighbourhood improved adult physical and mental health and subjective wellbeing over 10-15 years, despite no change in average economic status^64^. Moving to Opportunity was able to capitalize on the fact that public policy decisions are interconnected – it is not just health policies that influence mental health, substance use and other public health outcomes, but also economic, housing and criminal justice policies, among others^65^. Rapid growth in mental health and substance-use research over the past decade, as well as appeals from researchers and advocates to apply the findings in policy and practice have not yet bridged the divide between what is known and what is done^66,67^. The intricacies of ensuring evidence-based health policy are not entirely understood^68^, but a few effective practices are being used. Advocacy organizations such as the National Alliance on Mental Illness have become trusted sources of digestible research findings^69^. Carefully planned links between researchers and decision makers – an approach increasingly encouraged by funders of mental health and substance-use research – can also be effective^69^. Such links often involve collaboration among researchers, government agencies, advocates and provider institutions to synchronize research activities with policies, health-care demands and community priorities, and to engage key stakeholders in the identification of pressing research questions and the use of study findings. In this way, policymakers have become partners in the research enterprise, helping researchers to understand what information is needed for developing or updating policies, making investment decisions, expanding access to care, improving care quality and monitoring system-level change over time. The long-term goal is that these partnerships will mobilize political will, inform policy development, and shed light on the essentials of shaping science-informed mental health and substance-use policies.

Inclusion of mental health as an explicit priority in the post-2015 development agenda (such as that included in the UN Open Working Group on Sustainable Development Goals, 2015; https://sustaina-bledevelopment.un.org/content/documents/7891TRANSFORMING%20OUR%20WORLD.pdf) provides an opportunity to mobilize the requisite political will and resources at several levels so that this ambitious agenda for research and capacity building can be realized. Lessons learned from the positive health impact as a result of Millennium Development Goals 4, 5 and 6 illuminate how multisector and multilevel cohesion of effort and commitments are powerful levers for advancing health in low-resource settings, and an opportunity for the broad community of stakeholders and advocates to improve care for individuals living with mental health and substance-use disorders.

## COLLABORATIVE CAPACITY BUILDING

New commitments and additional resources will be needed to rapidly cultivate the in-country research capacity needed to respond to the global disease burden of mental health and substance-use disorders^70^. The most culturally sensitive, scientifically and ethically sound, and locally relevant research requires investigators who best understand and live among the populations that they study. Funding initiatives such as the Fogarty International Center’s Global Brain and Nervous System Disorders Across the Lifespan programme (http://www.fic.nih.gov/Programs/Pages/Brain-Disorders.aspx), the NIMH’s Collaborative Hubs for International Research in Mental Health (www.nimh.nih.gov/about/organization/gmh/globalhubs/index.shtml) as well as Grand Challenges Canada’s Global Mental Health granting programme (http://www.grandchallenges.ca/grand-challenges/global-mental-health/) that explicitly structure research capacity building into grant requirements, provide exemplary platforms to test and ultimately to systematize innovative strategies for training, mentorship and building a research culture and other infrastructural support for research in LMICs.

In addition, collaborative capacities to advance the mental health and substance-use research agenda must be developed. Capacity building in knowledge management is also integral to packaging accrued evidence so that it is accessible to policymakers and mental health technology specialists in LMICs. Platforms for knowledge sharing (for example, the Mental Health Innovation Network, http://mhinnovation.net/; and GHD Online, http://www.ghdonline.org/) can promote scientific discovery and help to harmonize the mental health and substance-use disorder research goals, processes and tools, and to catalyse the translational potential of research to policy and programmes^71^. Moreover, these platforms are needed to build and consolidate the community of advocates, consumers, investigators, clinicians and policymakers united in their commitment to mitigate the suffering associated with mental health and substance-use disorders, eliminate their attendant stigma, diminish their social and economic burdens, and erase the social and health disparities perpetuated by poor access to high-quality mental health care.

## CONCLUSIONS

The formidable and rising health, economic and social burdens associated with mental health and substance-use disorders call for the prioritization of research that can inform a global response – through the development and enhancement of preventive and therapeutic strategies, health-system strengthening and policymaking – to alleviate suffering and stem the associated economic and social consequences of unmet needs. Indeed, the potential synergies among breakthroughs in basic neuroscience, epidemiological methods and implementation science, as well as the mobilization of resources and political will have generated optimism and catalysed a commitment to act among policymakers, advocates and the scientific community. Although the increase in mental health research initiatives over the past two decades are encouraging for the future challenges remain and patterns of progress have been inconsistent. We find, for example, that although response to the growing burden of depression in LMICs has led to an increase in the number of studies on effectiveness of treatments, delivery methods and task shifting to provide access to care for all populations, we do not see this same trajectory of efforts to address substance-use disorders. This occurs with the background of growing substance-use problems globally. Approaches to address substance-use disorders in LMICs are still limited, fragmented and not well vetted scientifically or culturally. On an optimistic note, the draft Social Development Goals to be passed by the UN General Assembly in September 2015 recognise mental health as integral to health and mental health is explicitly included within universal health coverage; in addition, the UN General Assembly will hold a special session on drugs in 2016. These developments have symbolic and substantive importance, and auger well for mental health within the Global Health agenda in the coming years.

## Supplementary Material

1

## Figures and Tables

**Table 1:** Summary of priority research areas and guiding principles for approaches to reduce the global burdens of mental and substance-use disorders.

Research domain	Priority areas
**Epidemiology**	• Refine approaches to diagnostic assessment across diverse local contexts by enhancing their local validity within universal frameworks, and promoting and evaluating their clinical utility for non-specialist health professionals and other health workers, including in a wide variety of health-care and community-based settings • Improve accuracy of estimates of health and related social and economic burdens • Advance understanding of local contextual factors that mediate and moderate risk, course of illness, and recovery for mental and substance-use disorders by identifying synergies among co-morbid mental health and substance-use disorders, and medical conditions relating to risk, illness trajectories and treatment outcomes: understanding context-specific impacts of social adversities and local resources that promote resilience: illuminating social vulnerabilities that impede access to care; examining and comparing population-specific phenotypic variants of major mental health and substance-use disorders to enhance understanding of genetic, developmental, environmental, social and cultural contributions to aetiology, course and presentation • Evaluate health, social and economic benefits of general health, mental health and substance-use disorder therapeutic intervention, prospectively across the life course and the next generation
**Basic sciences**	• Advance understanding of gene-environment interactions with the scientific benefits that variation across diverse contexts provide • Identify moderators of course of illness, therapeutic response, remission, relapse and recovery
**Health-care delivery and implementation**	• Optimize effectiveness of task-sharing models for community-based case-finding and treatment delivery • Understand and resolve barriers to integration of mental health and substance-use disorder assessment and treatment in primary care settings and in non-health platforms (for example, schools, housing and the criminal justice system) • Mitigate barriers to care access • Develop and improve coordinated approaches to strategic preventive interventions, monitoring and targeted treatments over the life course and across disorders • Evaluate successful delivery models at scale and their adaptation to diverse local contexts • Advance understanding of factors that mediate and moderate successful care delivery at scale
**Translational research and health policy**	• Illuminate ‘sideways’ impacts of economic, housing, criminal justice and education policies on mental health and substance-use disorders • Develop evidence-based strategies that accelerate the uptake of mental health research findings by policymakers
**Guiding principles**	**Rationale for approach**
**Responsive to exigent needs related to burden of disease and associated treatment gaps**	• Consistent with the broad global health agenda of reducing health burdens, research recommendations focus on a globalizing framework and leveraging strategy that can bring effective interventions to a larger scale • This pragmatic focus should not eclipse the complementary relevance and importance of extending research into comparative differences in mental and substance-use disorders across diverse populations and social contexts
**Context driven**	• Diversity across local social, economic, environmental, cultural and political contexts of health and health care has profound implications for risk, resilience, presentation, consumer demand, therapeutic outcomes, and impacts of mental and substance-use disorders: however, these factors remain incompletely understood • Engagement of local knowledge is an essential complement to universalizing frameworks and the aggregate global empirical base in developing a fully contextualized understanding and optimization of local responses to mental health and substance-use disorders as well as in formulating and pursuing the most salient local research priorities
**Integrative across primary and specialty clinical care domains and the life course**	• Mental disorders, substance-use disorders and medical conditions are frequently co-morbid and thus development, evaluation and implementation of delivery models that enhance coordination and integration of care – across disorders, conditions and the life course – are likely to reduce the burden and impacts of these disorders
**Inter-sectoral**	• Improved links between scientific evidence and policymaking can enable and accelerate translation from research to practice • Cross-sector planning can promote efficiencies and achievement of health sector goals by anticipating necessary preliminary or parallel actions in finance, education and other sectors
**Capacity building**	• Research activities should encompass an agenda and plan for in-country capacity building to grow the global research work force, extend the quality and reach of scientific enquiry, and promote a health equity agenda • Capacity-building should be bidirectional and collaborative, when possible, to tap complementary expertise and experience in research, clinical practice, implementation, policy and other relevant dimensions that will advance the local and global mental health agenda
