# Supplementary figures and images for: Global research challenges and opportunities for mental health and substance-use disorders
Source: Nature. Author manuscript; Available in PMC 2019 Feb 8. (PMC6368440; doi:10.1038/nature16032)

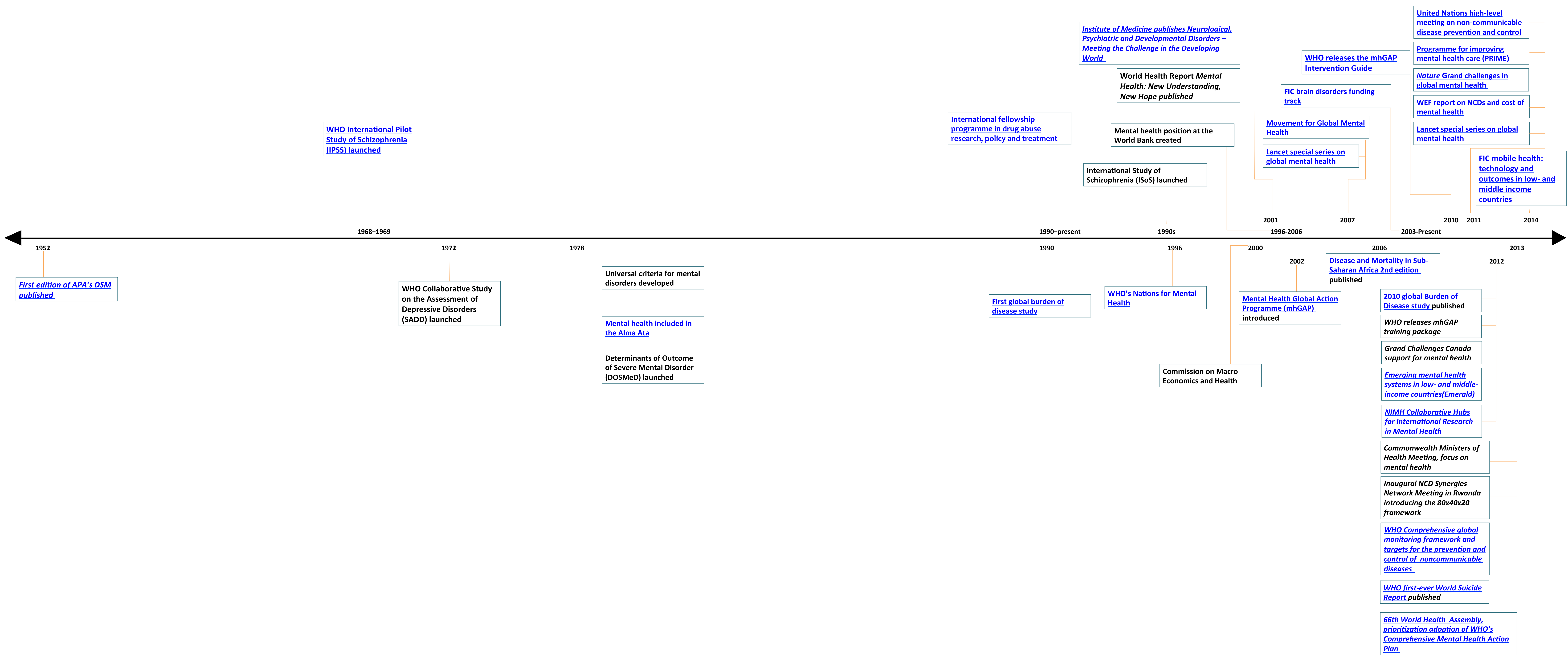

Supplement: 1 [file NIHMS994603-supplement-1.pdf]
